# Non-contiguous finished genome sequence and description of *Bartonella senegalensis* sp. nov.

**DOI:** 10.4056/sigs.3807472

**Published:** 2013-06-06

**Authors:** Oleg Mediannikov, Khalid El Karkouri, Georges Diatta, Catherine Robert, Pierre-Edouard Fournier, Didier Raoult

**Affiliations:** 1 URMITE, Aix-Marseille Université, Marseille, France, and Campus commun UCAD-IRD d'Hann, Dakar, Senegal

**Keywords:** *Bartonella senegalensis*, genome, Senegal, soft tick, *Ornithodoros sonrai*

## Abstract

*Bartonella senegalensis* sp. nov. strain OS02^T^ is the type strain of *B. senegalensis* sp. nov., a new species within the genus *Bartonella*. This strain, whose genome is described here, was isolated in Senegal from the soft tick *Ornithodoros sonrai*, the vector of relapsing fever. *B. senegalensis* is an aerobic, rod-shaped, Gram-negative bacterium. Here we describe the features of this organism, together with the complete genome sequence and its annotation. The 1,966,996 bp-long genome contains 1,710 protein-coding and 46 RNA genes, including 6 rRNA genes.

## Introduction

*Bartonella* is the only genus of the family *Bartonellaceae* of *Alphaproteobacteria*. To date, 29 *Bartonella* species have been validly published [1,2], and many isolates have yet to be described. These bacteria are facultative intracellular pathogens, many of which infect erythrocytes [3]. At least 13 *Bartonella* species are associated with human diseases. *B. bacilliformis*, *B. quintana* and *B. henselae*, are relatively common human pathogens and cause Carrión’s disease, trench fever and cat scratch fever, respectively. Different species of *Bartonella* are also associated with chronic bacteremia and/or endocarditis, bacillary angiomatosis, peliosis hepatitis, retinitis, uveitis, and myocarditis [4].

The epidemiological cycle of bartonellae consists of a reservoir host, which is a vertebrate with a chronic intravascular infection and sustained bacteremia, and a vector (usually a blood-sucking arthropod such as fleas, sandflies or lice) that transfers the bacteria from the reservoir to a susceptible host. *Bartonella* species are typically associated with a specific primary host; *e.g*., *B. henselae* is commonly found in domestic and wild felids all over the world, including Africa [5-7], whereas *B. bacilliformis* is human-specific. Animal hosts of bartonellae include dogs, rabbits, coyotes, foxes, cattle, deer, elk and multiple rodent species [6,8-10]. For most pathogenic bartonellae (except *B. bacilliformis* and *B. quintana*), humans are accidental (secondary) hosts [6].

In 2003, La Scola *et al.* proposed a multilocus sequence analysis based on 4 genes and one intergenic spacer as a tool for the description of new *Bartonella* species [11]. Among these genetic markers, two, *i.e.*, *gltA* and *rpoB*, were particularly discriminatory, with new *Bartonella* isolates considered as new species if they exhibit <96.0% and <95.4% sequence identity with other validly published species for the 327- and 825-bp fragments of the *gltA* and *rpoB* genes, respectively [2,11-13]. This strategy of combining sequences from several genes, usually housekeeping genes, is congruent with the “gold-standard” DNA–DNA reassociation for several bacterial genera [14].

In this study, we used La Scola’s criteria and described the genome sequence as well as main phenotypic characteristics of strain OS02^T^. Here, we present a summary classification and a set of features for *B. senegalensis* sp. nov. strain OS02^T^ together with the description of the complete genomic sequence and annotation. These characteristics support the definition of the species *B. senegalensis*.

## Classification and features

Fifteen adult *Ornithodoros sonrai* soft ticks were collected in 2008 from rodent burrows in the Soulkhou Thissé village (a rural village in the Guinean-Sudanian zone in Senegal) as part of a prospective study on tick-borne relapsing fever in West Africa. Ticks were preserved at room temperature for 40 days without feeding prior to further testing. The isolation of *Bartonella* strains from ticks was performed as described previously [15] and the results will be reported elsewhere. Strain OS02 (Table 1) was obtained in June 2009 from a single tick following a 7-day incubation at 37°C in 5% CO_2_-enriched atmosphere on Columbia agar (BioMerieux, Marcy l'Etoile, France).

**Table 1:** Classification and general features of *Bartonella senegalensis* strain OS02^T^.

**MIGS ID**	**Property**	**Term**	**Evidence code^a^**
		Domain *Bacteria*	TAS [16]
		Phylum *Proteobacteria*	TAS [17]
		Class *Alphaproteobacteria*	TAS [3,18]
	Current classification	Order *Rhizobiales*	TAS [19,20]
		Family *Bartonellaceae*	TAS [21-23]
		Genus *Bartonella*	TAS [21,23-26]
		Species *Bartonella senegalensis*	IDA
		Type strain OS02^T^	IDA
	Gram stain	Negative	IDA
	Cell shape	Rod	IDA
	Motility	Non-motile	IDA
	Sporulation	Non-sporulating	IDA
	Temperature range	Mesophilic	IDA
	Optimum temperature	37°C	IDA
MIGS-6.3	Salinity	Growth in BHI medium + 5% NaCl	IDA
MIGS-22	Oxygen requirement	Aerobic	IDA
	Carbon source	Unknown	IDA
	Energy source	Unknown	IDA
MIGS-6	Habitat	Tick gut	IDA
MIGS-15	Biotic relationship	Facultative intracellular	IDA
	Pathogenicity	Unknown	
	Biosafety level	3	
MIGS-14	Isolation	Soft tick *Ornithodoros sonrai*	IDA
MIGS-4	Geographic location	Senegal	IDA
MIGS-5	Sample collection time	May 2009	IDA
MIGS-4.1	Latitude	14°03'N	IDA
MIGS-4.2	Longitude	15°31'W	IDA
MIGS-4.3	Depth	~ 0.5 m under surface	IDA
MIGS-4.4	Altitude	45 m above sea level	IDA

^a^ Evidence codes - IDA: Inferred from Direct Assay; TAS: Traceable Author Statement (*i.e*., a direct report exists in the literature); NAS: Non-traceable Author Statement (*i.e*., not directly observed for the living, isolated sample but based on a generally accepted property for the species or anecdotal evidence). Evidence codes come from the Gene Ontology project [27]. If the evidence is IDA, then the property was directly observed for a live isolate by one of the authors or an expert mentioned in the acknowledgements.

**Figure 1:**
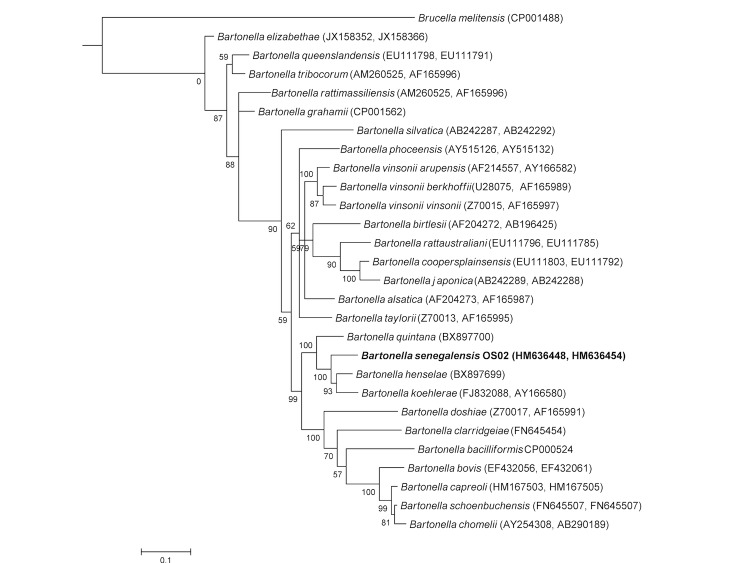
Phylogenetic tree highlighting the position of *Bartonella senegalensis* strain OS02^T^ relative to other type strains within the genus *Bartonella*. Concatenated *gltA* and *rpoB* sequences were aligned using CLUSTALW and phylogenetic inferences obtained using Bayesian phylogenetic analysis [31] with the TOPALi 2.5 software (Biomathematics and Statistics Scotland, Edinburgh, UK) with the integrated MrBayes application [32] with the GTR+Г substitution model. GenBank accession numbers are indicated in parentheses as (*gltA*, *rpoB*). Numbers at the nodes are bootstrap values obtained by repeating the analysis 100 times to generate a majority consensus tree. There were a total of 1,044 positions in the final dataset. The scale bar indicates a 10% nucleotide sequence divergence.

In addition to *gltA* and *rpoB* partial gene sequencing, we also sequenced the intergenic transcribed spacer (ITS) and the 16S rRNA and *ftsZ* genes as previously described [11,26,28-30]. Strain OS02 ^T^ exhibited nucleotide sequence similarities of 85.7, 99.3, 94.5, 94.9 and 93.6% for the ITS, 16S rRNA, *ftsZ*, *gltA* and *rpoB* genes, respectively, with *B. henselae*, the phylogenetically closest validly named *Bartonella* species (Figure 1).

**Figure 2:**
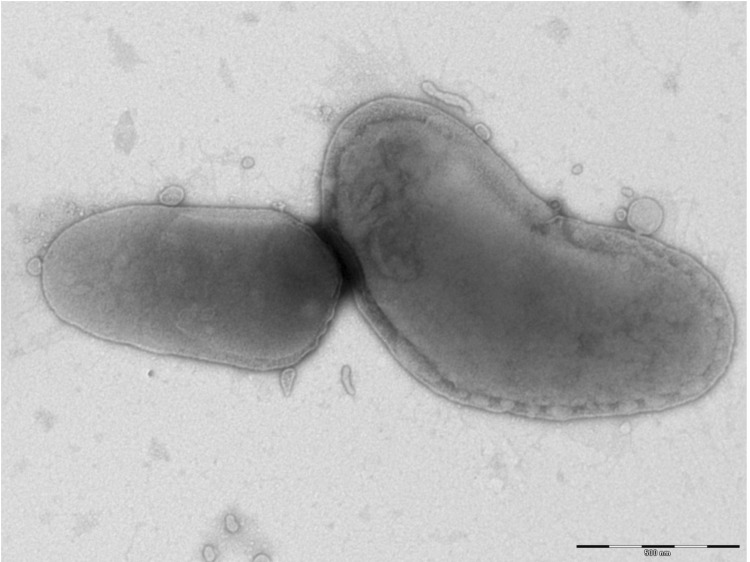
Transmission electron micrograph of *B. senegalensis* strain OS02^T^, using a Morgagni 268D (Philips) transmission electron microscope at an operating voltage of 60 kV. The scale bar represents 500 nm.

Different growth temperatures (32, 37, 42°C) were tested. Growth occurred only at 37°C in 5% CO_2_. Colonies were gray, opaque and 0.5 mm to 1 mm in diameter on blood-enriched Columbia agar. A motility test was negative. Cells grown on agar are Gram negative and have a mean length and width of 1,254.4±329.3 nm and 533.3±100.5 nm, respectively, by electron microscopy (Figure 2). No flagella or pili were observed.

Strain OS02^T^ exhibited neither catalase nor oxidase activity. Biochemical characteristics were assessed using an Anaerobe Identification Test Panel AN MicroPlate™ (Biolog Inc., Hayward, CA, USA). None of 95 biochemical tests available (including D-mannose, D-fructose and D-galactose) were positive. Similar profiles were previously observed for other *Bartonella* species [13].

**Figure 3:**
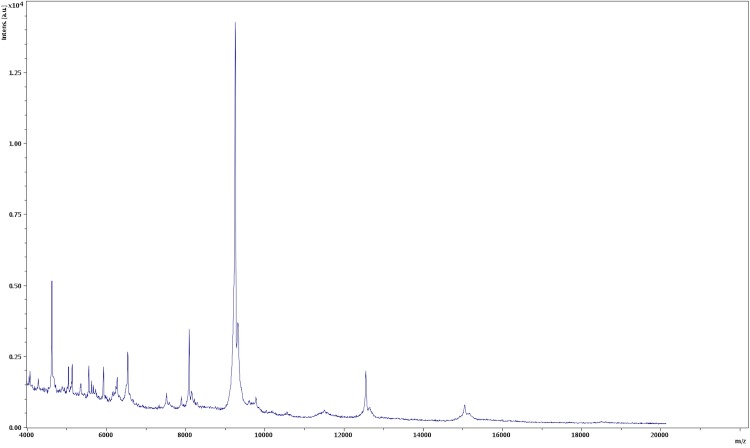
Reference mass spectrum from *B. senegalensis* strain OS02^T^. Spectra from 5 individual colonies were compared, and a reference spectrum was generated.

Matrix-assisted laser desorption/ionization time-of-flight (MALDI-TOF) mass spectrometry protein analysis was carried out as previously described [33]. Five isolated colonies of strain OS02^T^ were deposited as individual spots on the MALDI target plate. Each smear was overlaid with 2 μL of matrix solution (a saturated solution of alpha-cyano-4-hydroxycinnamic acid) in 50% acetonitrile/2.5% trifluoroacetic acid, and allowed to dry for five minutes. Measurements were performed with a Microflex spectrometer (Bruker). The five OS02^T^ spectra were imported into the MALDI BioTyper software (version 2.0, Bruker) and analyzed by standard pattern matching (with default parameter settings) against the main spectra of 4,613 bacteria in the BioTyper database and of 19 *Bartonella* species in our own database. The identification method included the *m/z* from 3,000 to 15,000 Da. For every spectrum, a maximum of 100 peaks were taken into account and compared with the spectra in the database. For strain OS02^T^, the scores obtained were always below 1.5 (a score < 1.7 did not enable an identification), suggesting that our isolate was not a member of a known species. We added the spectrum from strain OS02^T^ to the database (Figure 3).

## Genome sequencing information

### Genome project history

The organism was selected for sequencing on the basis of the similarity of its 16S rRNA, ITS, *ftsZ*, *gltA* and *rpoB* to other members of the genus *Bartonella*. Nucleotide sequence similarity levels of these genes suggested that strain OS02^T^ represents a new species in the genus *Bartonella*. A summary of the project information is shown in Table 2. The GenBank accession number is CALV00000000, and the entry consists of 99 contigs (≥200 bp) and 9 scaffolds (>1,500 bp). Table 2 shows the project information and its association with MIGS version 2.0 compliance.

**Table 2:** Project information

**MIGS ID**	**Property**	**Term**
MIGS-31	Finishing quality	High-quality draft
MIGS-28	Libraries used	One paired-end 3-kb library
MIGS-29	Sequencing platforms	454 GS FLX Titanium
MIGS-31.2	Fold coverage	30×
MIGS-30	Assemblers	Newbler version 2.5.3
MIGS-32	Gene calling method	Prodigal
	Genbank ID	CALV00000000
	Genbank Date of Release	August 17, 2012
MIGS-13	Project relevance	Biodiversity of *Ornithodoros sonrai* flora

### Growth conditions and DNA isolation

*B. senegalensis* sp. nov. strain OS02^T^ (DSM 23168; CSUR B623) was grown on 5% sheep blood-enriched Columbia agar at 37°C in a 5% CO_2_ atmosphere. Four Petri dishes were spread and resuspended in 3×100 μl of G2 buffer (EZ1 DNA Tissue kit, Qiagen). A first mechanical lysis was performed by glass powder on the Fastprep-24 device (Sample Preparation system; MP Biomedicals, USA) using 2×20-second cycles. DNA was then treated with 2.5 μg/μL lysozyme (30 minutes at 37°C) and extracted through the BioRobot EZ 1 Advanced XL (Qiagen). The DNA was then concentrated and purified on a Qiamp kit (Qiagen). The yield and concentration were measured by the Quant-it Picogreen kit (Invitrogen) on the Genios Tecan fluorometer at 130.4 ng/μl.

### Genome sequencing and assembly

DNA (5 μg) was mechanically fragmented on a Hydroshear device (Digilab, Holliston, MA, USA) with an enrichment size of 3-4 kb. The DNA fragmentation was visualized using the Agilent 2100 BioAnalyzer on a DNA labchip 7500 with an optimal size of 3.475 kb. The library was constructed according to the 454 GS FLX Titanium paired-end protocol. Circularization and nebulization were performed and generated a pattern with an optimum at 641 bp. After PCR amplification over 17 cycles followed by double size selection, the single-stranded paired-end library was then quantified with the BioAnalyzer on a DNA labchip RNA pico 6,000 at 323 pg/μL. The library concentration equivalence was calculated as 9.24E+08 molecules/μL. The library was stored at -20°C until further use.

The library was clonally amplified with 1 cpb and 1.5 cpb in 4 and 3 emPCR reactions, respectively, with the GS Titanium SV emPCR Kit (Lib-L) v2 (Roche). The yields of the 1 cpb and 1.5 cpb emPCR were determined to be 3.08% and 8%, respectively. After amplification, 790,000 beads from the 2 emPCR conditions were loaded on a ¼ region on the GS Titanium PicoTiterPlate PTP Kit 70×75 and sequenced with the GS FLX Titanium Sequencing Kit XLR70 (Roche). The run was analyzed on the cluster through the gsRunBrowser and Newbler assembler (Roche). A total of 200,243 passed filter wells were obtained and generated 57.62 Mb of DNA sequence with an average length of 287 bp. The passed filter sequences were assembled using Newbler with 90% identity and 40 bp for overlap requirements. The final assembly identified 9 scaffolds and 63 large contigs (≥1,500 bp), generating a genome size of 1.98 Mb, which corresponds to 29.10× equivalent genome.

### Genome annotation

Open reading frames (ORFs) were predicted using PRODIGAL [34] with default parameters, but predicted ORFs were excluded if they spanned a sequencing gap region. The predicted bacterial protein sequences were searched against the GenBank database [35] using BLASTP and the Clusters of Orthologous Groups (COG) database using COGNITOR [36]. The prediction of RNA genes, *i.e.*, rRNAs, tRNAs and other RNAs, was performed using the RNAmmer [37] and ARAGORN [38] algorithms. The transmembrane helices and signal peptides were identified using TMHMM [39] and SignalP [40], respectively.

## Genome properties

**Figure 4:**
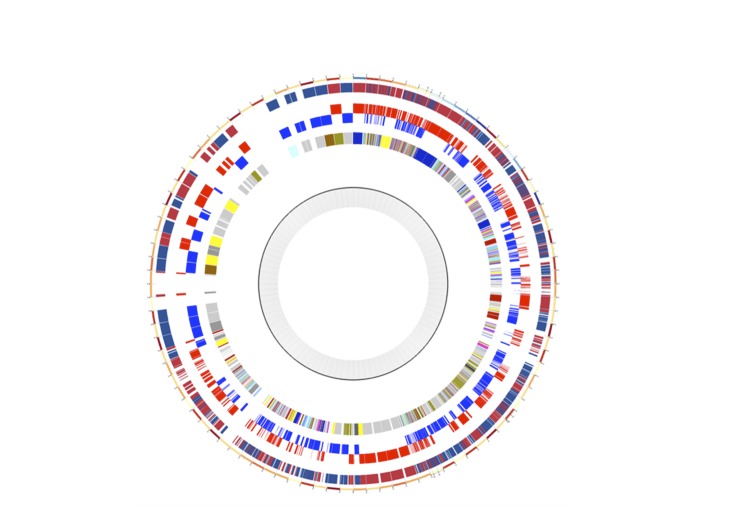
Circular map of the genome. From the outside in: genes on both forward and reverse strands, genes on the forward strand (red circle), genes on the reverse strand (blue circle) and genes colored by COG categories.

The genome is 1,966,996 bp long (one chromosome, no plasmids) with a 38.6% G+C content (Table 3, Figure 4). Of the 1,756 predicted genes, 1,710 were protein-coding genes, and 46 were RNAs (2 rRNA operons and 40 tRNA genes). A total of 997 genes (58.3%) were assigned a putative function. The remaining genes were annotated as either hypothetical proteins or proteins of unknown functions. The distribution of genes into COGs functional categories is presented in Table 4. The properties and the statistics of the genome are summarized in Tables 3 and 4.

**Table 3:** Nucleotide content and percentage of the genome

**Attribute**	**Value**	**% of total^a^**
Genome size (bp)	1,966,996	100
DNA coding region (bp)	1,488,480	75.7
DNA G+C content (bp)	760,125	38.6
Total genes	1,756	100
RNA genes	46	2.6
Protein-coding genes	1,710	97.4
Protein with predicted function	997	58.3
Genes assigned to COG	1,425	83
Genes with peptide signal	81	47.3
Genes with transmembrane helices (≥3)	178	10.4

^a^The total is based on either the size of the genome in base pairs or the total number of protein coding genes in the annotated genome.

**Table 4:** Number of genes associated with the 25 general COG functional categories^†^.

**Code**	**Value**	**%age**	**Description**
J	143	8.36	Translation
A	0	0	RNA processing and modification
K	76	4.44	Transcription
L	98	5.73	Replication, recombination and repair
B	0	0	Chromatin structure and dynamics
D	22	1.29	Cell cycle control, mitosis and meiosis
Y	0	0	Nuclear structure
V	10	0.58	Defense mechanisms
T	38	2.22	Signal transduction mechanisms
M	89	5.20	Cell wall/membrane biogenesis
N	3	0.18	Cell motility
Z	0	0	Cytoskeleton
W	6	0.35	Extracellular structures
U	68	3.98	Intracellular trafficking and secretion
O	76	4.44	Posttranslational modification, protein turnover and chaperones
C	81	4.74	Energy production and conversion
G	60	3.51	Carbohydrate transport and metabolism
E	124	7.25	Amino acid transport and metabolism
F	43	2.51	Nucleotide transport and metabolism
H	57	3.33	Coenzyme transport and metabolism
I	42	2.46	Lipid transport and metabolism
P	74	4.33	Inorganic ion transport and metabolism
Q	15	0.88	Secondary metabolites biosynthesis, transport and catabolism
R	183	10.70	General function prediction only
S	117	6.84	Function unknown
X	449	26.26	Not in COGs

^†^The total is based on the total number of protein coding genes in the annotated genome.

## Insights from the genome sequence

Compared to *B. henselae* strain Houston (GenBank accession number NC_005956), its closest phylogenetic neighbor, *B. senegalensis* strain OS02^T^ had a larger genome (1,966,996 and 1,931,047 bp, respectively), more genes (1,756 and 1,491 genes, respectively) and a higher G+C content (38.6 and 38%, respectively). The protein-coding genes present in *B. senegalensis* but absent or split in *B. henselae* included multidrug-efflux transporter, membrane protein formate-tetrahydrofolate ligase, formate-tetrahydrofolate ligase, glycoside hydrolase family 3-like, glycoside hydrolase family 3-like, putative major facilitator superfamily, SAM-dependent methyltransferases, resolvases, toxin-antitoxin modules, transposases, ubiquinol-cytochrome C reductase, *LeuA2*, and phage proteins, as well as several hypothetical proteins.

## Conclusion

On the basis of phylogenetic and genotypic analyses, we formally propose the creation of *Bartonella senegalensis* sp. nov., which contains strain OS02^T^. This bacterium was isolated in Senegal.

### Description of *Bartonella senegalensis* sp. nov.

*Bartonella senegalensis* (se.ne.ga.len′sis. N.L. fem. adj. *senegalensis* referring to Senegal, the African country that is home to the *Ornithodoros sonrai* tick from which the type strain was isolated).

Colonies are opaque, grey and 0.5 to 1.0 mm in diameter on blood-enriched Columbia agar. Cells are rod-shaped without flagellae. Length and width are 1,254.4±329.3 nm and 533.3±100.5 nm, respectively. Growth is only obtained at 37°C. Cells stain Gram-negative, are non-endospore-forming, and are non-motile. Catalase and oxidase activities are absent. No biochemical activity is observed using the Anaerobe Identification Test Panel AN MicroPlate.

The ITS, 16S rRNA, *ftsZ*, *rpoB* and *gltA* genes, and draft genome sequences are deposited in GenBank under accession numbers HM636451, HM636442, HM636445, HM636454, HM636448 and CALV00000000, respectively. The genome is 1,966,996 bp long and contains 1,710 protein-coding and 46 RNA genes, including 6 rRNA genes. The G+C content is 38.6%. The type strain OS02^T^ (DSM 23168, CSUR B623) was isolated from an *O. sonrai* soft tick collected in a rodent burrow in a rural village in Senegal.
